# FE65 as a link between VLDLR and APP to regulate their trafficking and processing

**DOI:** 10.1186/1750-1326-7-9

**Published:** 2012-03-19

**Authors:** Sonya B Dumanis, Kelly A Chamberlain, Yoo Jin Sohn, Young Jin Lee, Suzanne Y Guénette, Toshiharu Suzuki, Paul M Mathews, Daniel TS Pak, G William Rebeck, Yoo-hun Suh, Hee-Sae Park, Hyang-Sook Hoe

**Affiliations:** 1 Department of Neuroscience, Georgetown University Medical Center, 3970 Reservoir Road NW, Washington, DC 20057-1464, USA; 2 Department of Neurology, Georgetown University Medical Center, 3970 Reservoir Road NW, Washington, DC 20057-1464, USA; 3 Department of Pharmacology, Georgetown University Medical Center, 3970 Reservoir Road NW, Washington, DC 20057-1464, USA; 4 Laboratory of Neuroscience, Graduate School of Pharmaceutical Science, Hokkaido University, Sapporo, Japan; 5 Nathan Kline Institute for Psychiatric Research, Orangeburg, New York, USA; 6 Department of Pharmacology, College of Medicine, Neuroscience Research Institute, MRC, Seoul National University, Seoul, South Korea; 7 Hormone Research Center, School of Biological Sciences and Technology, Chonnam National University, Gwangju 500-757, Republic of Korea; 8 Genetics and Aging Research Unit, Mass General Institute for Neurodegenerative Disease, Harvard Medical School, Charlestown, MA; 9

**Keywords:** FE65, VLDLR, APP trafficking

## Abstract

**Background:**

Several studies found that FE65, a cytoplasmic adaptor protein, interacts with APP and LRP1, altering the trafficking and processing of APP. We have previously shown that FE65 interacts with the ApoE receptor, ApoER2, altering its trafficking and processing. Interestingly, it has been shown that FE65 can act as a linker between APP and LRP1 or ApoER2. In the present study, we tested whether FE65 can interact with another ApoE receptor, VLDLR, thereby altering its trafficking and processing, and whether FE65 can serve as a linker between APP and VLDLR.

**Results:**

We found that FE65 interacted with VLDLR using GST pull-down and co-immunoprecipitation assays in COS7 cells and in brain lysates. This interaction occurs via the PTB1 domain of FE65. Co-transfection with FE65 and full length VLDLR increased secreted VLDLR (sVLDLR); however, the levels of VLDLR C-terminal fragment (CTF) were undetectable as a result of proteasomal degradation. Additionally, FE65 increased cell surface levels of VLDLR. Moreover, we identified a novel complex between VLDLR and APP, which altered trafficking and processing of both proteins. Furthermore, immunoprecipitation results demonstrated that the presence of FE65 increased the interaction between APP and VLDLR *in vitro *and *in vivo*.

**Conclusions:**

These data suggest that FE65 can regulate VLDLR trafficking and processing. Additionally, the interaction between VLDLR and APP altered both protein's trafficking and processing. Finally, our data suggest that FE65 serves as a link between VLDLR and APP. This novel interaction adds to a growing body of literature indicating trimeric complexes with various ApoE Receptors and APP.

## Background

FE65 and FE65-like (FE65L or FE65L1) proteins are cytoplasmic adaptor proteins that possess two phosphotyrosine binding domains (PTB1 and PTB2) and one WW binding domain. FE65 is primarily found in the CNS and is highly expressed in neurons of the hippocampus, cerebellum, thalamus, and brainstem nuclei in the adult mouse brain [1]. Several studies have shown that FE65 can form a stable, transcriptionally active complex with AICD (APP intracellular domain) in heterologous gene reporter systems [2-8], although the full range of gene targets is still unknown. FE65 is functionally linked to cellular motility and morphology and actin dynamics through binding of its WW domain to the actin-binding protein Mena [9,10]. Interestingly, FE65 and FE65L double knockout mice exhibit defects similar to triple APP knockout (APP tKO): lissencephaly and selected axonal projection defects [11].

The PTB2 domain of FE65 interacts with the NPXY motif of amyloid precursor protein (APP) [12-14] and this interaction mediates APP trafficking both *in vitro *and *in vivo *[13,15]. For example, in H4 neuroglioma cells, the induction of hFE65L increased the ratio of mature to total APP levels and increased secreted APPα (sAPPα) threefold [13]. Similar results were obtained in Madin-Darby Canine Kidney (MDCK) cells where overexpression of FE65 led to increased translocation of APP to the cell surface, increased secreted APPα, and increased Aβ secretion, [16]. In contrast to the H4 and MDCK cells, overexpression of full-length FE65 strongly decreased secreted APPα and APP C-terminal fragment (CTF) in CHO cells [17,18]. Overexpressing human FE65 in a Thy-1 APP transgenic mouse model also resulted in decreased Aβ accumulation in the cerebral cortex and decreased levels of APP CTF [14]. Therefore, it is unclear how FE65 could modulate APP trafficking and processing.

The PTB1 domain of FE65 interacts with ApoE receptors, including LRP1 and ApoER2, via the ApoE receptor's NPXY motif [17,19]. Moreover, FE65 acts as a functional linker between LRP1 and APP [20,21]. Overexpression of FE65 increased sAPP in LRP+/+ mouse fibroblasts; however, no significant effect on APP processing exists in LRP-/- fibroblasts, suggesting the effect of FE65 on APP processing is LRP dependent [20]. In a recent study, we have shown that a similar tripartite complex is formed between APP, FE65, and ApoER2 and that LRP1 may be competing with ApoER2 for FE65 binding sites [17]. This complex results in altered processing of both APP and ApoER2. Overexpression of FE65 led to a significant increase in secreted ApoER2, secreted ApoER2 CTF, and cell surface levels of ApoER2 in COS7 cells [17]. Whether FE65 can interact with other ApoE receptors, affecting receptor trafficking and processing, is unknown.

In the present study, we demonstrated a novel interaction between FE65 and VLDLR (very low density lipoprotein receptor) using a GST pull-down assay in brain lysates. Co-immunoprecipitation studies indicated that there was also a complex formed between APP and VLDLR, which is increased in the presence of FE65 *in vitro *and *in vivo*. This data suggests that FE65 acts as a linker between VLDLR and APP. Moreover, we found that these interactions modulate APP and VLDLR trafficking and processing.

## Results

### FE65 interacts with VLDLR

**Figure 1:**
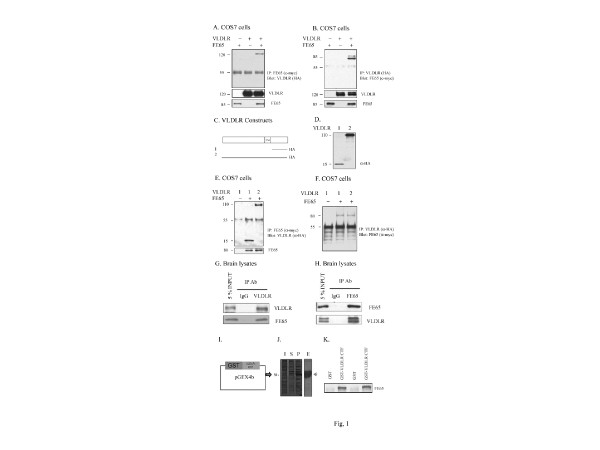
**FE65 interacts with VLDLR in COS7 cells and brain lysates**. **A**. COS7 cells were transiently transfected with VLDLR (HA-tagged on the C terminal) and FE65 (myc-tagged on the C terminal), or each construct alone with empty vector. Cell lysates (200 ug) were immunoprecipitated with anti-myc and the precipitate was probed with anti-HA. FE65 co-precipitated with VLDLR in COS7 cells (lane 3, upper panel). Cell lysates (20 ug) were probed for VLDLR and FE65 to demonstrate consistent levels of expression across each condition (middle and lower panel). **B**. COS7 cells were transfected with VLDLR (HA-tagged) and FE65 (myc-tagged), or each construct alone. Cell lysates (200 ug) were immunoprecipitated with anti-HA and the precipitate was probed with anti-myc. VLDLR co-precipitated with FE65 in COS7 cells (lane 3, upper panel). Cell lysates (20 ug) were probed for VLDLR and FE65 to demonstrate consistent levels of expression across conditions (middle and lower panel). **C**. VLDLR deletion construct expressing the CTF of VLDLR with an HA tag was generated. **D**. COS7 cells were transiently transfected with VLDLR or VLDLR CTF constructs. Cell lysates (20 ug) were probed for anti-HA to verify the expression of constructs. E-F. COS7 cells were transiently transfected with full length VLDLR and empty vector, full length VLDLR and FE65 or VLDLR CTF and FE65. **E**. Cell lysates (200 ug) were immunoprecipitated with anti-myc and the precipitate was probed with anti-HA. VLDLR CTF and full length VLDLR co-precipitated with FE65 in COS7 cells (lane 2 and 3, upper panel). Cell lysates (20 ug) were probed for FE65 to demonstrate consistent levels of expression across each condition (lower panel). **F**. Cell lysates (200 ug) were immunoprecipitated with anti-HA and the precipitate was probed with anti-myc. FE65 immunoprecipitated with both VLDLR constructs consistent with the experiment in **E**. **G**. Mouse brain lysates (100 ug) were immunoprecipitated with 5F3 (VLDLR antibody) or Ig G and probed with anti-VLDLR (upper panel) and anti-FE65 (lower panel). **H**. Mouse brain lysates (100 ug) were immunoprecipitated with anti-FE65 or IgG and probed with anti-FE65 (upper panel) and anti-VLDLR (lower panel). **I**. Schematic of recombinant GST-VLDLR CTF **J**. The recombinant GST-VLDLR CTF was expressed in *E.coli *strain BL21, using the pGEX system as indicated. The GST fusion proteins were then purified using glutathione-agarose beads (Sigma), in accordance with the manufacturer's instructions. (I, total cell extract of induced cells; S, supernatant of sonicated extracts of induced cells (soluble recombinant protein); P, Pellet of sonicated extracts of induced cells (insoluble recombinant protein); **E**, Purified VLDLR CTF proteins). **K**. Recombinant GST-VLDLR CTF proteins were immobilized onto GSH-agarose. Wild-type brain lysates were then subjected to GST-pull-down experiments with either immbolized GST (lane 1, 3) or immobilized GST-VLDLR CTF (lane 2, 4) and probed for FE65.

We used co-immunoprecipitation experiments to test whether FE65 interacted with VLDLR. COS7 cells were transfected with VLDLR and empty vector, VLDLR and FE65, or FE65 and empty vector. Full-length VLDLR co-precipitated with FE65 and was not detectable in the absence of FE65 (Figure 1A). Western blot analysis of COS7 cell extracts confirmed that levels of VLDLR and FE65 were consistent across transfections (Figure 1A). We also performed the reverse experiment, which showed that full-length FE65 co-precipitated with VLDLR and was not detectable in the absence of VLDLR (Figure 1B).

To test whether VLDLR CTF interacted with FE65, we transfected COS7 cells with full length VLDLR and empty vector, full length VLDLR and FE65, or VLDLR CTF and FE65 (Figure 1C, D), and performed co-immunoprecipitations. We found that FE65 co-precipitated with both full length VLDLR and VLDLR CTF (Figure 1E). Consistent with these findings, the reverse experiment resulted in co-precipitation of full length VLDLR and VLDLR CTF with FE65 in COS7 cells (Figure 1F).

We then examined whether there was a physical association between FE65 and VLDLR *in vivo*. To test this, we performed co-immunoprecipitations from whole brain lysates, using anti-5F3 to recognize VLDLR or a nonspecific IgG as a negative control. Immunoprecipitation of VLDLR resulted in the co-precipitation of FE65 (Figure 1G). In the reverse experiment, we performed co-immunoprecipitation from whole brain lysates using anti-FE65 and then probed with anti-5F3. We found that FE65 co-immunoprecipitated with both the mature and immature forms of VLDLR in brain lysates (Figure 1H). Overall, these results suggest that VLDLR interacts with FE65 both *in vitro *and *in vivo*. To further examine whether VLDLR interacts with FE65, we incubated wild-type brain lysates with purified immobilized GST or GST-VLDLR CTF protein and probed for FE65 (Figure 1 I-K). We found that VLDLR CTF interacted with FE65 *in vivo*. No signal was detected in lanes of brain lysates incubated with GST alone.

### FE65 co-localizes with VLDLR in primary hippocampal neurons

**Figure 2:**
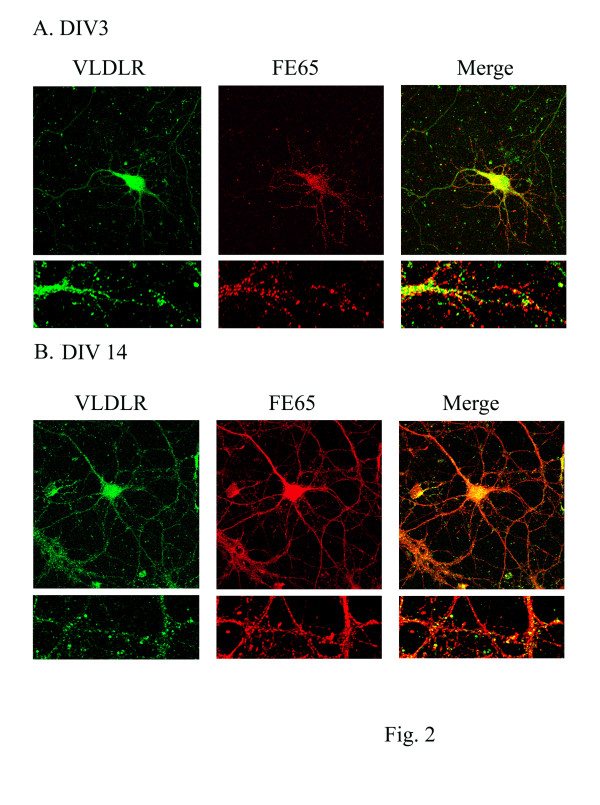
**FE65 co-localizes with VLDLR in primary hippocampal neurons**. **A**. After 3 days in culture, primary hippocampal neurons were fixed and immunostained for 5F3 (recognizing VLDLR) and anti-FE65. Antibodies were detected with Alexa Flour 488 anti-mouse antibody (in green for VLDLR) and Alexa Fluor 555 anti-rabbit antibody (in red for FE65), imaged with a confocal laser-scanning microscope (63X). Co-localization of VLDLR and FE65 appears yellow as vesicular staining in the right panel. **B**. After 14 days in culture, primary hippocampal neurons were fixed and immunostained for 5F3 (recognizing VLDLR) and anti-FE65. Antibodies were detected with Alexa Flour 488 anti-mouse antibody (in green for VLDLR) and Alexa Fluor 555 anti-rabbit antibody (in red for FE65), observed with a confocal laser-scanning microscope (63X). Co-localization of VLDLR and FE65 appears yellow as vesicular staining in the right panel, consistent with Figure 2A.

To test whether endogenous FE65 co-localizes with VLDLR during early neuronal development, primary hippocampal neurons (DIV 3) were fixed and immunostained with anti-5F3 and anti-FE65 antibodies. VLDLR and FE65 immunoreactivities were strong in the cell body and punctuate throughout neuronal processes (Figure 2A). The immunostainings overlapped suggesting that VLDLR co-localized with FE65 within the cell bodies and partially co-localized in neuronal processes (Figure 2A).

To test whether FE65 and VLDLR can still co-localize during the peak of synaptogenesis, primary hippocampal neurons (DIV 14) were fixed and immunostained with anti-5F3 and anti-FE65 antibodies. Interestingly, FE65 expression was up-regulated on DIV 14 compared to DIV3, consistent with previous findings [1]. Moreover, VLDLR and FE65 immunoreactivity was strong in the cell body and punctuate throughout neuronal processes with partial co-localizations, consistent with what we observed on DIV 3 (Figure 2B).

### VLDLR interacts with the PTB1 domain of FE65

**Figure 3:**
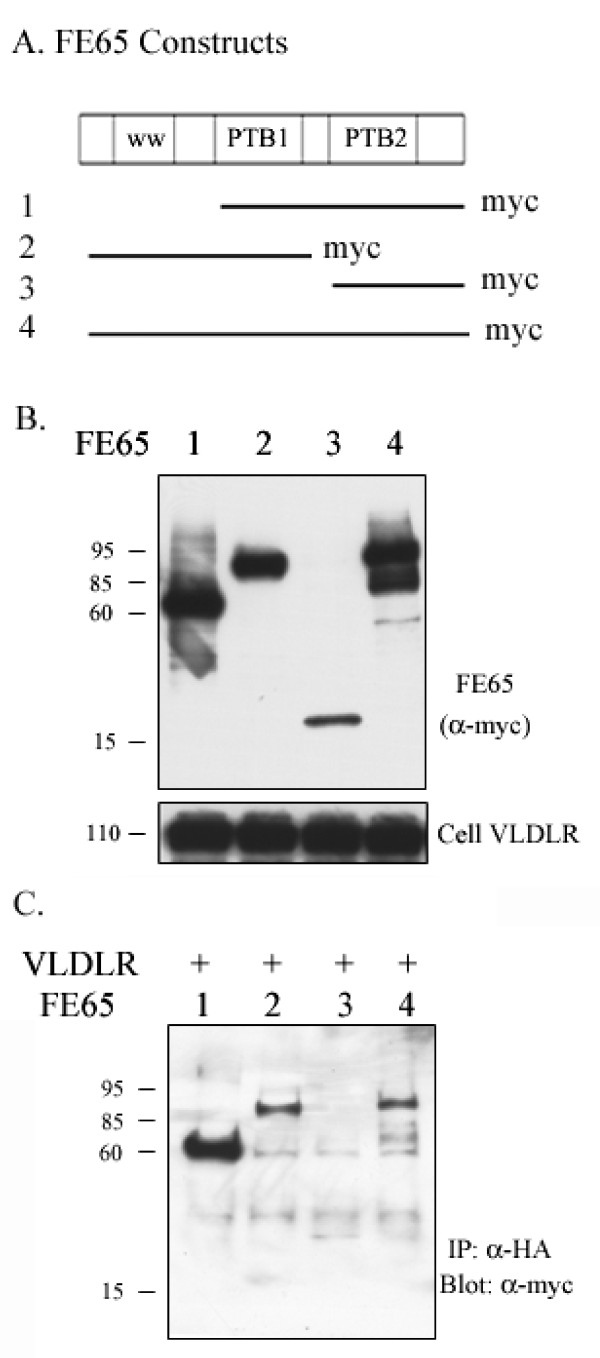
**VLDLR interacts with the PTB1 domain of FE65 A**. Constructs of FE65 with C-terminal Myc tags containing PTB1 and PTB2 (construct 1, ~65 KD), WW and PTB1 (construct 2, ~85 KD), only PTB2 (construct 3, ~15 KD), and full length FE65 (construct 4, ~95 KD). **B**. Western blot analysis showing comparable expression levels of the different FE65 constructs (upper panel). The VLDLR immunoblotting (bottom panel) showed similar expression levels of VLDLR in all transfected cells. **C**. COS7 cells were transfected with plasmids expressing different FE65 constructs (indicated along the top of the panel) and full length VLDLR. Cell lysates (200 ug) were immunoprecipitated with anti-HA antibody (for VLDLR) and probed with an anti-myc antibody (for FE65). FE65 constructs containing the PTB1 domain (constructs 1, 2, 4) co-immunoprecipitated with VLDLR, but not constructs containing only the PTB2 domain (construct 3).

To determine which domain of FE65 interacts with VLDLR, COS7 cells were co-transfected with full length VLDLR and FE65 deletion constructs containing a c-terminal myc tag (Figure 3A). Each FE65 construct resulted in protein expression at the anticipated sizes, as determined by western blot (Figure 3B, upper panel). VLDLR was expressed to similar levels in all transfected cells (Figure 3B, lower panel). Immunoprecipitation with an anti-HA antibody (for VLDLR) and probing with an anti-myc antibody (for FE65) resulted in VLDLR immunoprecipitation with all three FE65 constructs containing the PTB1 domain, but not the FE65 containing only the PTB2 domain construct (Figure 3C, upper panel). Interestingly, VLDLR interacted strongly with the FE65 construct lacking the WW domain (construct #1) compared to full length FE65 (construct #4) and the FE65 construct containing only the WW and PTB1 domains (construct #2) (Figure 3C). However, the FE65 WW domain alone does not co-precipitate with VLDLR (data not shown). Since it has been shown that the WW and PTB domains of FE65 can interact with each other [3], the FE65 WW domain may induce conformational changes in full length FE65 which reduce the exposure of the FE65 PTB1 domain for interaction with VLDLR.

We conducted an additional experiment to ensure that the lack of co-immunoprecipitation between VLDLR and the FE65 containing only the PTB2 domain was not due to the decreased expression level of the FE65 PTB2 domain (construct #3) in cell lysates. To test this, we used a different set of FE65 deletion constructs, which have a GFP c-terminal tag. COS7 cells were co-transfected with full length VLDLR-myc and GFP, VLDLR-myc and FE65 PTB2-GFP (construct #1), or VLDLR-myc and full length FE65-GFP (construct #2) (Additional file 1: Figure S1A). VLDLR and each FE65 construct resulted in similar protein expression in all transfected cells (Additional file 1: Figure S1B). Immunoprecipitation with an 5F3 antibody (for VLDLR) and probing with an anti-GFP antibody (for FE65) resulted in full length FE65 immunoprecipitation with the VLDLR but the FE65 construct containing only the PTB2 domain did not. Consistent with these findings, the reverse experiment resulted in co-precipitation of VLDLR with the full but not with the truncated PTB2 construct (Additional file 1: Figure S1B).

### FE65 affects VLDLR processing

**Figure 4:**
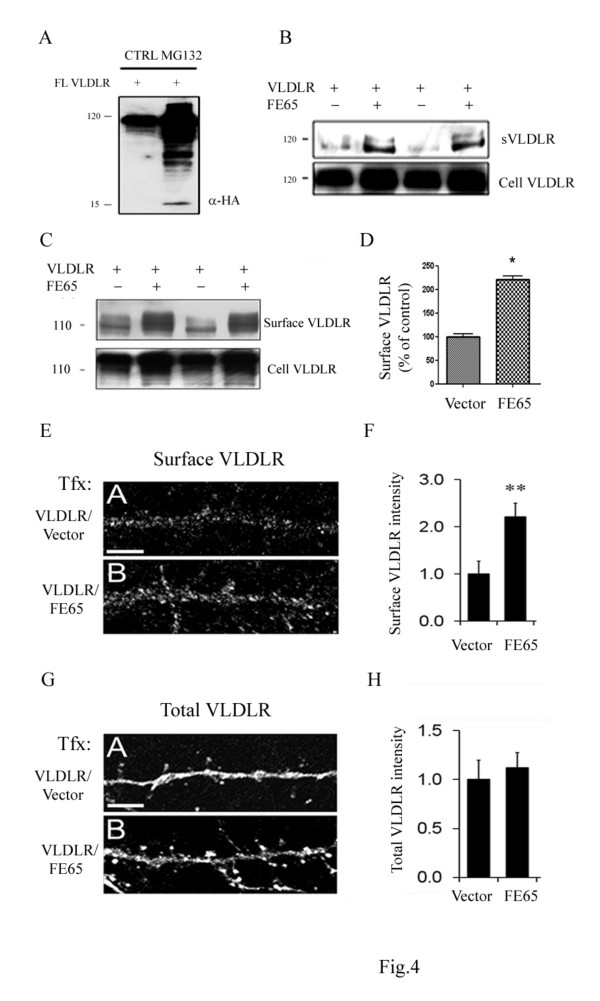
**FE65 increases cell surface levels of VLDLR A**. COS7 cells were transiently transfected with full length VLDLR for 24 hours. After transfection, cells were grown in serum-free media and incubated with MG132 (10 uM) (lane 2) or DMSO (lane 1) as control for 24 hr. Cell lysates (20 ug/lane) were then probed with anti-HA antibody. Consistent with previous findings, VLDLR CTFs were only detectable in the presence of MG132. **B**. COS7 cells were transiently transfected with VLDLR and empty vector or VLDLR and FE65. Secreted VLDLR was measured in conditioned media (14 ul) with 5F3 antibody. Total VLDLR and VLDLR CTF were measured in cell lysates (20 ug/lane) with anti-HA. FE65 increased levels of sVLDLR, suggesting that FE65 affects VLDLR processing. **C**. COS7 cells were transfected with VLDLR and vector (lanes 1, 3) or VLDLR and FE65-myc (lanes 2, 4). Cell surface proteins were biotin-labeled, isolated with avidin-beads, and immunoblotted with the 5F3 antibody for VLDLR detection. Full length FE65 increased surface levels of VLDLR (upper blot). Expression of VLDLR across all transfections in shown in the lower blot. **D**. Quantification of surface levels of VLDLR normalized to control in (**C**). FE65 increases cell surface levels of VLDLR by 118% (n = 4, *p *< 0.001). **E**. Cultured hippocampal neurons (DIV14) were transfected with GFP, VLDLR, and empty vector (upper panel) or GFP, VLDLR, and FE65 (lower panel). Surface VLDLR was measured with the 5F3 antibody by immunofluorescence of live cells. **F**. Quantification of cell surface VLDLR intensity in neuronal processes in (**E**). The cell surface staining in neuronal processes showed a 1.2 fold increase in cell surface levels of VLDLR with FE65 (n = 10, *p *< 0.05). **G**. Cultured hippocampal neurons (DIV14) were transfected with GFP, VLDLR, and empty vector (upper panel) or GFP, VLDLR, and FE65 (lower panel). Total levels of VLDLR were measured with the 5F3 antibody by immunofluorescence after permeabilization of cells. **H**. Quantification of total VLDLR intensity in neuronal processes in (**G**).

Our previous studies have shown that VLDLR undergoes α- and γ- secretase cleavage similar to APP and ApoER2 [22]. Because VLDLR CTFs were undetectable with overexpression of full length VLDLR, we hypothesized that VLDLR CTF may undergo proteasome degradation. To test this possibility, COS7 cells were transfected with full length VLDLR and treated with the proteasomal inhibitor, MG132 (10 uM) or vehicle (10% DMSO) for 24 hours. We found that VLDLR CTFs were detectable when full length VLDLR-transfected cells were treated with MG132 (Figure 4A). Interestingly, there was also a large increase in full length VLDLR suggesting that both VLDLR CTFs and full length VLDLR undergo proteasomal degredation.

To test whether FE65 could modulate VLDLR processing *in vitro*, COS7 cells were transfected with VLDLR-HA and empty vector or VLDLR-HA and FE65, and the levels of sVLDLR, total VLDLR, and VLDLR CTF were measured. Co-transfection of FE65 increased sVLDLR (Figure 4B, upper panel) and had no effect on total VLDLR levels in COS7 cells (Figure 4B, lower panel). VLDLR CTFs were still undetectable in the presence of FE65. These data suggest that FE65 may regulate VLDLR processing.

### FE65 increases cell surface levels of VLDLR

To test whether FE65 could affect VLDLR trafficking, we transfected COS7 cells with full length VLDLR and empty vector or full length VLDLR and FE65 for 24 hours. Cell surface proteins were biotinylated, isolated with avidin beads, and immunoblotted for VLDLR. We found that FE65 significantly increased cell surface levels of VLDLR by 118% (Figure 4C, D, n = 4/condition). To verify our findings, we conducted live cell surface staining by overexpressing GFP, VLDLR and empty vector or GFP, VLDLR and FE65 in primary hippocampal neurons (DIV 14-16). FE65 increased cell surface levels of VLDLR by 120%, a 1.2 fold increase, in primary hippocampal neurons (Figure 4E, F, *p *< 0.05; n = 10). However, total VLDLR protein level was unchanged in the presence of FE65, consistent with our previous *in vitro *and *in vivo *data (Figure 4G, H). Thus, two independent assays suggest that FE65 can modulate cell surface expression of VLDLR.

### FE65 and VLDLR CTF translocate into the nucleus

**Figure 5:**
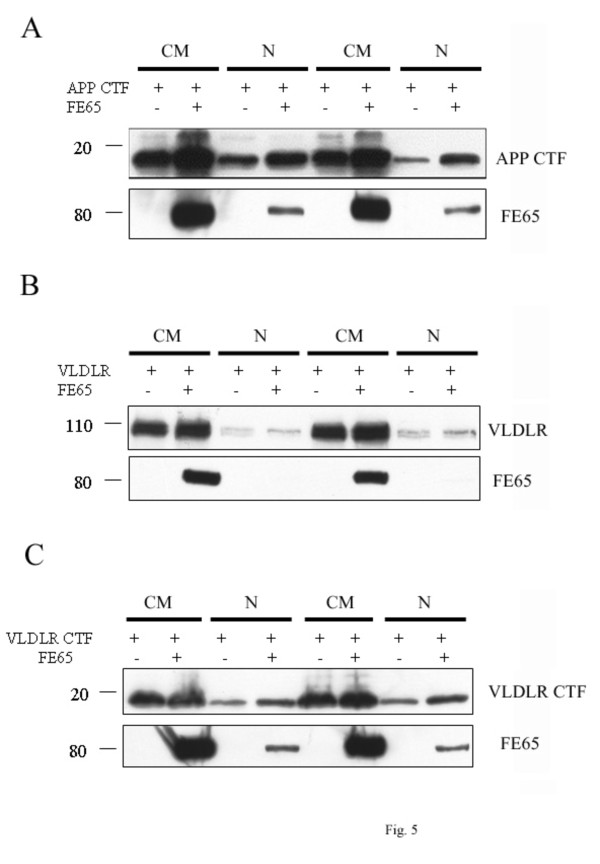
**FE65 and VLDLR CTF complex translocates into the nucleus**. **A**. COS7 cells were transfected with APP CTF and vector or APP CTF and FE65 and cellular fractionation was conducted. Cytoplasmic/Membrane (CM) and nuclear (N) fractions were immunoblotted with the C1/6.1 antibody (for APP) and the anti-myc antibody (for FE65). **B**. COS7 cells were transfected with full length VLDLR and vector or full length VLDLR and FE65 and cellular fractionation was conducted. Cytoplasmic/Membrane (CM) and nuclear (N) fractions were immunoblotted with the 5F3 antibody (for VLDLR) and the anti-myc antibody (for FE65). **C**. COS7 cells were transfected with VLDLR CTF and vector or VLDLR CTF and FE65 and cellular fractionation was conducted. Cytoplasmic/Membrane (CM) and nuclear (N) fractions were immunoblotted with the anti-HA antibody (for VLDLR CTF) and the anti-myc antibody (for FE65).

Several studies have shown that FE65 and the cytoplasmic domain of APP form a complex and translocate into the nucleus in COS7 and H4 cells [2,3,23,24]. Consistent with previous findings, we observed that APP CTF was present in nuclear fractions when co-expressed with FE65 compared to controls (Figure 5A). We then examined whether FE65 could also translocate VLDLR CTFs to the nucleus. To test this, we transfected COS7 cells with full length VLDLR or VLDLR CTF with either FE65 or empty vector. We found that full length VLDLR and FE65 were present in the cytosol/membrane fractionation, but were not present in the nucleus (Figure 5B). Similar to APP CTF and FE65 complex, VLDLR CTF and FE65 were expressed in both the cytosolic/membrane and in the nucleus (Figure 5C).

### VLDLR interacts with APP and affects processing of both proteins

**Figure 6:**
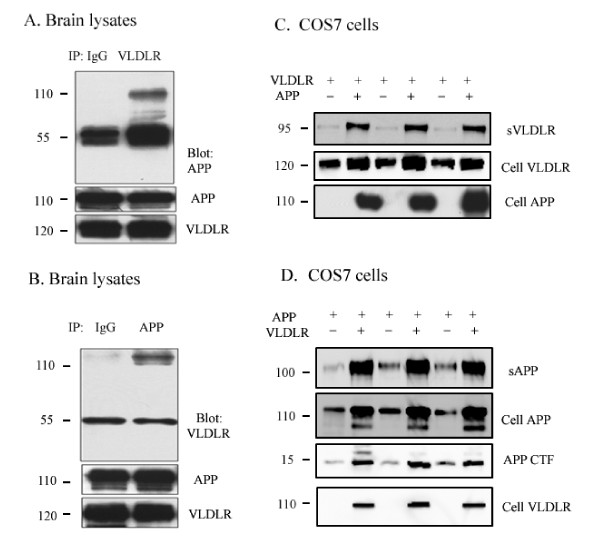
**VLDLR interacts with APP and affects the processing of both proteins**. **A**. Mouse brain lysates (100 ug) were immunoprecipitated with 5F3 (VLDLR antibody) or IgG antibody and probed with the C1/6.1 antibody for APP. **B**. Mouse brain lysates (100 ug) were immunoprecipitated with C1/6.1 (APP antibody) or IgG antibody and probed with 5F3 antibody for VLDLR. **C**. COS7 cells were transfected with VLDLR-myc and vector (lanes 1, 3, 5) or VLDLR-myc and APP-HA (lanes 2, 4, 6). Secreted VLDLR was measured in conditioned media with 5F3 antibody, and total levels of VLDLR and APP were measured in cell lysates with anti-myc antibody and C1/6.1, respectively. **D**. COS7 cells were transfected with APP and vector (lanes 1, 3, 5) or APP and VLDLR (lanes 2, 4, 6). Secreted APP was measured in conditioned media with 6E10 antibody, and total levels of APP and APP CTF were measured in cell lysates with C1/6.1. Total levels of VLDLR were measured in cell lysates with anti-myc antibody.

ApoE Receptors, including LRP1 and ApoER2, have been shown to interact with APP [17,19-21,25], and thus we wanted to investigate whether VLDLR can interact with APP. For this experiment, we performed co-immunoprecipitations from whole brain lysates using anti-VLDLR antibody or an anti-IgG antibody and probed for APP. We observed that APP co-precipitated with VLDLR *in vivo *(Figure 6A, upper panel). We also conducted the reverse experiment and found that VLDLR co-precipitated with APP (Figure 6B). APP and VLDLR were expressed to similar levels in all conditions (Figure 6A, B lower panels).

To examine the effect of APP on VLDLR processing, we transfected COS7 cells with full length VLDLR and empty vector or full length VLDLR and APP, and then the levels of sVLDLR, total VLDLR, VLDLR CTF, and total APP were measured. Co-transfection with APP resulted in increased sVLDLR and total VLDLR compared to empty vector (Figure 6C). However, VLDLR CTF levels remained undetectable.

Next, COS7 cells were transfected with APP and empty vector or APP and VLDLR in order to examine the effect of VLDLR on APP processing. VLDLR increased the levels of total APP, sAPPα and APP CTF (Figure 6D). These data suggest that the interaction between APP and VLDLR affects the metabolism of both proteins.

### VLDLR and APP affect cell surface expression of each other

**Figure 7:**
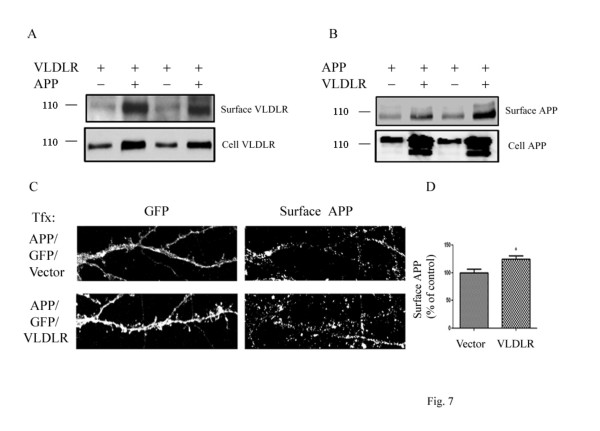
**The interaction between APP and VLDLR regulates the cell surface levels of both proteins**. **A**. COS7 cells were transfected with VLDLR and vector (lanes 1, 3) or VLDLR and APP (lanes 2, 4). Cell surface proteins were biotin-labeled, isolated with avidin-beads, and immunoblotted for VLDLR. APP increased surface levels of VLDLR (upper blot). **B**. COS7 cells were transfected with APP and vector (lanes 1, 3) or APP and VLDLR (lanes 2, 4). Cell surface proteins were biotin-labeled, isolated with avidin-beads, and immunoblotted for APP. VLDLR increased surface levels of APP (upper blot). **C**. Cultured hippocampal neurons (DIV14) were transfected with GFP, APP, and empty vector (upper panel) or GFP, APP, and VLDLR (lower panel). Surface APP was measured with an anti-APP antibody by immunofluorescence of live cells. **D**. Quantification of cell surface APP intensity in neuronal processes in (**C**). The cell surface staining in neuronal processes showed a 24% increase in APP by VLDLR (n = 10, *p *< 0.05).

We next examined whether APP alters cell surface expression of VLDLR. COS7 cells were transfected with VLDLR and empty vector or VLDLR and APP, and cell surface biotinylation was performed. We found that APP increased cell surface levels of VLDLR (Figure 7A). We also examined whether VLDLR can regulate cell surface expression of APP. COS7 cells were transfected with APP and empty vector or APP and VLDLR. We found that VLDLR increased cell surface levels of APP (Figure 7B). To further examine the effects of VLDLR on APP trafficking, primary hippocampal neurons were transfected with GFP, APP, and empty vector or GFP, APP, and VLDLR and live cell surface staining was conducted. Consistent with our findings, VLDLR significantly increased cell surface levels of APP by 24% (*p *< 0.05, n = 10) (Figure 7C, D).

### FE65 increases interaction between VLDLR and APP *in vitro *and *in vivo*

**Figure 8:**
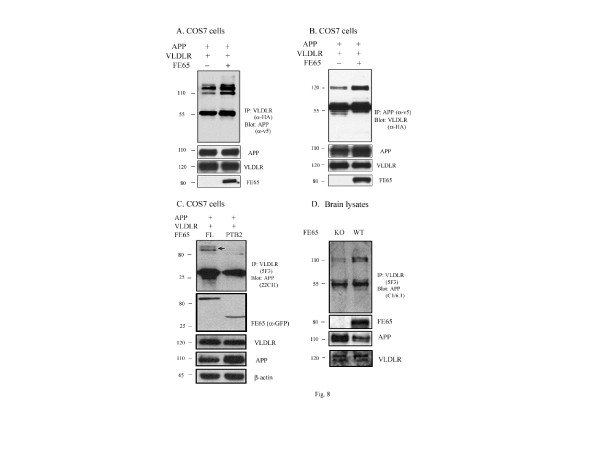
**FE65 increased co-precipitation of APP and VLDLR**. **A**. COS7 cells were transfected with APP-v5, empty vector, and VLDLR-HA or APP-v5, FE65-GFP, and VLDLR-HA. Cell lysates were immunoprecipitated with anti-HA antibody (for VLDLR) and probed with anti-V5 (for APP). Immunoblots of cell lysates showed similar levels of APP and VLDLR. **B**. COS7 cells were transfected with APP, empty vector, and VLDLR or APP, FE65, and VLDLR. Cell lysates were immunoprecipitated with anti-V5 antibody (for APP) and probed with anti-HA (for VLDLR). Immunoblots of cell lysates showed similar levels of APP and VLDLR. **C**. COS7 cells were transfected with APP, VLDLR and either full length FE65-GFP or FE65-PTB2 domain-GFP. Cell lysates were immunoblotted with anti- GFP antibody (for FE65 constructs), VLDLR, APP and β-actin was used as the loading control (lower panels). Cell lysates were immunoprecipitated with anti-5F3 (for VLDLR) and probed for anti-22C11 (for APP) (top panel). n = 3 per condition. **D**. Brain lysates from FE65 knockout mice and wild-type littermates were immunoprecipitated with anti-5F3 and immunobloted for anti-C1/6.1. Immunoblots for total levels of APP (C1/6.1), VLDLR (IIII) and FE65 are in the lower panels.

We and others have shown that FE65 forms tripartite complexes with APP and LRP1 or ApoER2, modulating the interaction of these proteins [17,20]. We investigated whether FE65 can affect the interaction between VLDLR and APP *in vitro*. COS7 cells were transfected with VLDLR, APP, and empty vector or VLDLR, APP, and FE65. Immunoprecipitation with an anti-VLDLR antibody and probing for APP revealed that FE65 increased the interaction between VLDLR and APP in COS7 cells (Figure 8A). In the reverse experiment, co-transfection with FE65 increased the association between APP and VLDLR (Figure 8B).

To verify whether FE65 can modulate the interaction between APP and VLDLR, we transfected COS7 cells with APP, VLDLR and either full length FE65 or FE65 PTB2 domain, which interacts with APP but not VLDLR. Cell lysates were immunoprecipitated with an anti-5F3 antibody (for VLDLR) and probed with an anti-22C11 antibody (for APP) (Figure 8C). We found that FE65 PTB2 domain construct significantly decreased the association between APP and VLDLR compared to full-length FE65 (Figure 8C). To examine whether FE65 can alter the association between APP and VLDLR *in vivo*, we immunoprecipitated VLDLR from brain lysates and found that an APP immunoreactive band was decreased in FE65 knockout brain lysates compared to wild-type littermates (Figure 8D). These data further demonstrate that FE65 is a linker between APP and VLDLR.Total levels of VLDLR were unchanged in FE65 knockout mice compared to wildtype littermates. (Figure 8D, lower panel). Interestingly, FE65 knockout mice had significantly increased total APP and APP CTFs compared to wild-type littermates (Figure 8D, Additional file 2: Figure S**2**). These data indicate that FE65 may also differentially regulate the processing of APP and VLDLR.

## Discussion

Previous studies have shown that FE65 interacts with ApoE receptors, LRP1 [19] and ApoER2 [17,25]. In the present study, we discovered a novel interaction between FE65 and VLDLR using GST pull-down and co-immunoprecipitation assays (Figure 1). We have previously shown that FE65 increased cell surface levels of ApoER2 *in vitro *[17]. In that same study, we found that FE65 increased sApoER2 and ApoER2 CTF in COS7 cells, while knockdown of FE65 caused decreased ApoER2 CTF *in vivo *[17]. However, whether FE65 can alter LRP1 trafficking and processing is unknown.

In this study, we examined the effects of FE65 on VLDLR trafficking and processing and found that FE65 increases VLDLR on the cell surface *in vitro *(Figure 4), similar to the effect of FE65 on ApoER2 trafficking [17]. In addition, FE65 increased sVLDLR, while total VLDLR remained unchanged in COS7 cells (Figure 4) and brain lysates (Figure 8D). Consistent with our previous findings [17], VLDLR CTF was undetectable without the presence of the proteasomal inhibitor MG132 when full length VLDLR was overexpressed. Additionally, we observed increased expression of full length VLDLR with MG132 treatment, suggesting that both VLDLR CTF and full length VLDLR may undergo proteasome degradation (Figure 4A). To further support our findings, a recent study demonstrated that the E3 ubiquitin Ligase IDOL targets the VLDLR receptor for degradation, specifically through the lysine residues adjacent to the NPXY motif [26].

Several studies have shown that the PTB2 domain of FE65 interacts with APP, thereby affecting its trafficking and processing in numerous cell lines [12-14,27-30]. These studies have differed in the observed effects of FE65 on APP processing. We found that FE65 increased sAPPα and decreased Aβ production in COS7 cells [17], perhaps by modulating APP trafficking. In contrast, we and others have shown that FE65 decreased sAPPα in CHO cells [17,18], suggesting that the effects in different cell types may be due to different interacting proteins. Guenette et al. examined the effect of FE65 on APP processing *in vivo *and found that total APP levels were unchanged in 3-4 month old FE65 knockout mice compared to wild-type littermates [11]. Interestingly, we observed that 13 month old FE65 knockout mice have an increase total APP and APP CTF compared to wild-type littermates (Additional file 1: Figure S1), suggesting that FE65 alters APP processing in an age-dependent manner.

Several studies have shown that FE65 complexes with APP CTF or AICD resulting in translocation of this complex, along with Tip60, to the nucleus where they likely participate in gene transcription events [2,3,23,26]. Also, over-expression of LRP1 intracellular domain (LRP ICD) and FE65 resulted in translocation of these proteins into the nucleus, which inhibited transcription activation mediated by the APP and FE65 complex [23]. However, whether the ApoER2 CTF and FE65 complex can translocate into the nucleus is unknown. Consistent with previous findings, we found that APP CTF and FE65 resulted in localization of the nuclear fraction (Figure 5A). Additionally, we observed that co-expression of FE65 and VLDLR CTF resulted in translocations of FE65 and VLDLR CTF in the nucleus (Figure 5C). This data suggest that similar to APP CTF and FE65, VLDLR CTF and FE65 translocate into the nucleus to play a role in gene transcription. It is possible that VLDLR CTF and FE65 may inhibit APP CTF/FE65 transcriptional activation, similar to LRP ICD [24]. Future studies are required to understand the biological significance of this translocation; genes may be preferentially regulated by VLDLR CTF and FE65 compared to APP CTF or LRP ICD and FE65.

Numerous studies have shown that the ApoE receptors interact with APP directly or indirectly through FE65 [17,19-21,25]; thus, we examined whether a similar interaction occurs between APP and VLDLR. We found that VLDLR co-precipitated with APP in brain lysates and vice versa, suggesting that these proteins may form a complex *in vivo *(Figure 6A, B). Several studies have shown that ApoE receptors including ApoER2, LRP1, LRP1B, SORL1 and LRAD3 regulate APP trafficking and processing [18,23,31-37]. For example, LRP1 and LRP1B have been directly linked to the formation of Aβ *in vitro *[18,31-33] and disruption of LRP1 and LRP1B with APP interaction leads to increased cell surface expression of APP and reduced Aβ production [34]. Overexpression of ApoER2 results in increased cell surface levels of APP, increased Aβ production, and a reduction in APP CTFs *in vitro *[35]. In contrast, our study has shown that ApoER2 significantly increased cell surface levels of APP, increased sAPPα, and decreased Aβ levels [22]. SORL1, another member of the ApoE receptor family, has also been implicated in APP trafficking [36]. Additionally, a recently discovered ApoE receptor, LRAD3, has also been shown to interact with APP and affect APP processing by decreasing sAPPα and increasing Aβ production [37]. Interestingly, FE65 does not interact with LRAD3 suggesting that there are multiple pathways by which ApoE receptors can influence APP processing and trafficking.

In the present study, we investigated whether VLDLR could also affect APP trafficking and processing. We found that full length VLDLR increased cell surface levels of APP (Figure 7B) as well as the levels of sAPPα and APP CTF in COS7 cells (Figure 6D). This is consistent with previous studies, which have found that retention of APP at the cell surface increases sAPPα production [38,39]. Conversely, we found that co-transfection of VLDLR with APP resulted in increased cell surface levels of VLDLR (Figure 7A) as well as levels of sVLDLR (Figure 6C), suggesting that the VLDLR/APP complex is retained at the cell surface where it can be cleaved by α-secretase. Surprisingly, co-expression of APP and VLDLR increased the total levels of both molecules (Figure 7A, B). Since we observed that full length VLDLR undergoes proteosomal degradation (Figure 4C), perhaps the interaction between APP and VLDLR slows down its proteosomal degradation resulting in increased total protein expression. Taken together, these data suggest that ApoE receptors associate with APP and these interactions regulate APP trafficking and processing and vice versa. However, whether this effect is due to a direct interaction or modulation by cytoplasmic adaptor proteins, such as FE65, is unknown.

We and others have shown that FE65 can functionally link APP with ApoEr2 and LRP and that this complex modulates both the ApoE receptor and APP trafficking and processing [17,18,31-33]. Here, we examined whether FE65 could form an intracellular link between APP and VLDLR and found that full length FE65 increased co-precipitation of APP with VLDLR *in vitro *(Figure 8A, B) and *in vivo *(Figure 8D), suggesting that FE65 serves as a link between APP and VLDLR. Interestingly, our recent study showed that ApoEr2 and LRP1 can compete for binding of FE65 [17] to alter APP trafficking and processing. Thus, it is possible that VLDLR may compete with other ApoE receptors to bind to FE65, and consequently alter APP trafficking and processing. Future studies will clarify under which conditions the ApoE receptors compete with FE65 and subsequently alter the regulation of APP.

What is the biological significance of this proposed complex? FE65 is known to interact with molecules important in actin remodeling through its WW domains [9], promoting the movement of neuronal growth cones and aiding in cell motility [10]. Mice lacking FE65, VLDLR, or ApoER2 display defects in neuronal migration [11,40-42]. FE65 has also been implicated in hippocampus-dependent learning and long-term potentiation [43]. Additionally, ApoER2, VLDLR, and APP knockout mice exhibit impaired learning and memory and LTP [44-47]. We and others have also demonstrated that ApoER2 and APP play an important role in dendritic spine formation [18,29,36,37,48,49]. Based on the literature and our findings, we hypothesize that the interaction of FE65, ApoE receptors, and APP could affect neuronal migration, learning and memory, as well as dendritic spine formation. To support our hypothesis, we observed that co-expression of FE65 and VLDLR altered the pattern of VLDLR immunostaining along the dendritic shaft and increased dendritic spine density compared to controls (Figure 2). We are currently pursuing these findings to understand whether a trimeric complex versus a dimeric complex is formed, which functions each is involved in, and how these protein complex's regulate the function of interest.

## Conclusions

In summary, we found that FE65 associates with VLDLR and alters its trafficking and processing. Additionally, the association of FE65 with VLDLR CTF can translocate into the nucleus similar to the APP CTF and FE65 complex. Moreover, FE65 enhances the interaction between VLDLR and APP, and this association affects the trafficking and processing of both proteins. This work demonstrates a novel complex between FE65, VLDLR, and APP, which helps elucidate the role of FE65 in regulating transmembrane proteins like ApoE receptors and APP in the CNS.

## Methods

### Vector construction

We generated C-terminal tagged myc and C-terminal tagged HA for full length VLDLR and C-terminal of VLDLR. Recombinant DNA sequences were confirmed by sequencing, and expression of correctly sized proteins was confirmed by Western blot.

### Cell lines and culture conditions

COS7 was maintained in Opti-MEM (Invitrogen) with 10% fetal bovine serum (FBS, Life Technologies, Inc.) in a 5% CO_2 _incubator. COS7 cells were transiently transfected with 0.5 -1 ug of plasmid in FuGENE6 (Roche) according to the manufacturer's protocol and cultured 24 h in DMEM containing 10% FBS. For co-transfections, cells were similarly transfected with 0.5-1 ug of each plasmid in Fugene 6 (Roche) and cultured 24 hr in DMEM with 10% FBS. After 24 hr the cells were transferred to Opti-MEM serum free media (Invitrogen) and treated with indicated compounds.

### Isolation of nuclei

For isolation of nuclear fraction, cells were harvested and 200 μl of ice-cold CER1 was added to the cell pellet, vortexed vigorously to fully resuspend the cell pellet. The tube was incubated on ice for 10 min, 11 μl of ice-cold CER II was added, vortexed for 5 sec, and centrifuged for 5 min (16, 000 × g, 5 min). Immediately after the supernatant (cytoplasmic/membrane extract) fraction was transferred, and the insoluble (pellet) fraction was resuspended in 100 ul of ice-cold NER. This was then vortexed for 15 sec, and returned to ice for continued vortexing for 15 sec every 10 min, for a total of 40 min. The sample was then centrifuged for 10 min (16, 000 × g, 10 min) and the supernatant (nuclear extract) fraction was immediately transferred.

### Antibodies

We used antibodies anti-HA (Abcam), anti-c-myc (Abcam), anti-22C11 (Chemicon), anti-V5 (Chemicon), and anti-FE65 (From Dr. Andre Goffinet). The anti-5F3 antibody was a kind gift of Dr. Dudley Strickland, the C1/6.1 antibody was a kind gift from Dr. Paul Matthew, and the VLDLR IIII antibody was a kind gift of Dr. Guojun Bu. For analysis of secreted APP, we used 6E10 (identifying sAPPα) (Signet).

### Quantification of VLDLR and APP proteolytic fragments

Secreted fragments were identified by western blot analysis of the media (sVLDLR, 5F3 antibody; sAPP, 6E10 antibody). CTF were measured by western blots of cell lysates (VLDLR CTF, myc antibody; APP CTF, C1/6.1 antibody).

### Culture and transfection of primary hippocampal neurons

Primary hippocampal neurons from embryonic day 18-19 Sprague-Dawley rats were cultured at 150 cells/mm^2 ^as described [38]. Neurons were transfected at 14 days *in vitro *(DIV) with GFP, APP-HA, VLDLR-Myc or empty vector by lipofectamine 2000 (Invitrogen) (2 μg DNA per well) according to manufacturers instructions. Transcription of each insert was driven by the CMV promoter.

### Biotin-labeled cell surface proteins

COS7 cells were transiently transfected with VLDLR and vector or VLDLR and FE65 in Fugene 6 (Roche) and cultured 24 hrs in DMEM containing 10% FBS. After 24 hr, cells were washed twice with PBS, and surface proteins were labeled with Sulfo-NHS-SS-Biotin 500 ul at 500 ug/ml PBS (Pierce) under gentle shaking at 4°C for 30 min. 50 ul of quenching solution was added to cells at 4°C, which were then washed twice with TBS. Cells were lysed in 500 ul lysis buffer, collected with a cell scraper, disrupted by sonication on ice, incubated for 30 min on ice, and clarified by centrifugation (10, 000 × g, 2 min). To isolate biotin-labeled proteins, lysate was added to immobilized NeutrAvidin TM Gel (50 ul) and incubated 1 hr at room temperature. Gels were washed five times with wash buffer and incubated 1 hr with SDS-PAGE sample buffer including 50 mM DTT. Elutions were analyzed by immunoblotting.

### Immunostaining and live cell surface staining

Hippocampal cultured neurons were fixed in methanol at -20°C for 10 min (for immunostaining of endogenous synaptic markers). Antibodies for immunostaining were incubated in GDB buffer (0.1% gelatin, 0.3% Triton X-100, 16 mM sodium phosphate pH 7.4, 450 mM NaCl).

Cell surface expression levels of VLDLR were performed as described [17]. Live neuronal cultures were briefly incubated (10 min) with the 5F3 antibody directed against extracellular N-termini of VLDLR (10 μg/mL in conditioned medium) to specifically label surface receptors, then lightly fixed for 5 min in 4% paraformaldehyde (non-permeabilizing conditions). After fixation, the surface-remaining antibody-labeled protein was measured with Alexa Fluor 555-conjugated anti-mouse secondary antibodies for 2 hr. Immunostaining was quantified using Metamorph analysis of immunostaining intensity or punctate number from Z-stacked images obtained with a Zeiss LSM510 confocal microscope. Surface localization of staining was also confirmed visually from these images.

### Co-immunoprecipitations

Brain Lysates from 13 month old FE65 knockout mice and wild-type littermate were homogenized in buffer containing 50 mm Tris-HCl, pH 8.0, 0.15 m NaCl, 1% Nonidet P-40, and phosphatase and protease inhibitors (IP Buffer). For immunoprecipitations, lysates were incubated overnight at 4°C with APP or VLDLR antibody and protein G-Sepharose beads (Amersham Biosciences). The precipitates were washed five times with lysis buffer and resuspended in SDS sample buffer.

### GST pull down assay

The recombinant GST or GST-VLDLR CTF protein was expressed in Escherichia coli BL21 strain, using the pGEX-4B system as previously described (Kim, et al 2011). The GST or GST-VLDLR CTF fusion protein was then purified using glutathione-agarose beads (Sigma), in accordance with the manufacturer's instructions. An equal amount of GST or GST-VLDLR CTF fusion protein was incubated overnight with brain lysates of wild-type mice (n = 3 per each condition). After incubation, protein-A agarose was added, and the samples were incubated for 3 hours at 4°C on a rotator. Following incubation, the beads were washed three times in ice-cold PBS and boiled with Laemmli sample buffer.

### Statistical analyses

Experiments were repeated a minimum of four times unless otherwise noted. Data were analyzed using t-tests with significance determined as p < 0.05. Descriptive statistics were calculated with StatView 4.1 and displayed as an expressed mean ± S.E.M.

## Abbreviations

VLDLR: Very Low Density Like Receptor; APP: Amyloid Precursor Protein; AICD: Amyloid Precursor protein intracellular domain; apoEr3: Apolipoprotein Receptor 2; LRP1: Lipoprotein receptor related protein 1; CTF: C-terminal Fragment; sVLDLR: secreted VLDLR; sAPP: secreted APP.

## Competing interests

The authors declare that they have no competing interests.

## Authors' contributions

Conceived and designed the experiments: HSH SBD Performed the experiments: SBD KAC YJS YJL H-SH. Analyzed the data: SBD KAC YJS YJL Contributed reagents/materials/analysis tools: DTS YHS PMM HSP TS SYG. Wrote the paper: SBD KAC HSH GWR. All authors read and approved the final manuscript.

## Supplementary Material

Additional file 1 **Figure S1. The FE65 PTB2 domain does not interact with VLDLR**. A. Constructs of FE65 with C-terminal GFP tags containing only PTB2 (construct #1) and full length FE65 (construct # 2). B. COS7 cells were transfected with VLDLR-myc and GFP, FE65 PTB2-GFP or full length FE65-GFP. Cell lysates (100 ug) were immunoprecipitated with 5F3 antibody (for VLDLR) and probed with an anti-GFP antibody (for FE65). Full length FE65 immunoprecipitates with VLDLR, but not the FE65 containing only the PTB2 domain (first panel). Conversely, cell lysates (100 ug) were immunoprecipitated with GFP (for FE65) and probed with an anti-myc (for VLDLR) (second panel). Western blot analysis showing comparable expression levels of the two FE65 constructs and VLDLR (third and fourth panel). Click here for file

Additional file 2 **Figure S2. FE65 Knockout mice have increased APP processing**. 13 month old FE65 knockout mice or wild-type littermates were immunoblotted with FE65, C1/6.1 (for APP), and β-actin. FE65 knockout mice had increased levels of full length APP and APP CTF compared to wild type littermates. Click here for file
