# Supplementary figures and images for: FE65 as a link between VLDLR and APP to regulate their trafficking and processing
Source: Mol Neurodegener. 2012 Mar 19;7:9. doi: 10.1186/1750-1326-7-9 (PMC3379943; doi:10.1186/1750-1326-7-9)

# A. FE65

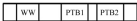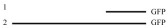

# B.

|               |   |   |   |
|---------------|---|---|---|
| VLDLR-myc     | + | + | + |
| FE65 PTB2-GFP | - | + | - |
| FL FE65-GFP   | - | - | + |

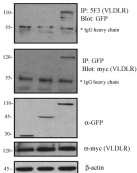

Supplement: Additional file 1 — Figure S1. The FE65 PTB2 domain does not interact with VLDLR. A. Constructs of FE65 with C-terminal GFP tags containing only PTB2 (construct #1) and full length FE65 (construct # 2). B. COS7 cells were transfected with VLDLR-myc and GFP, FE65 PTB2-GFP or full length FE65-GFP. Cell lysates (100 ug) were immunoprecipitated with 5F3 antibody (for VLDLR) and probed with an anti-GFP antibody (for FE65). Full length FE65 immunoprecipitates with VLDLR, but not the FE65 containing only the PTB2 domain (first panel). Conversely, cell lysates (100 ug) were immunoprecipitated with GFP (for FE65) and probed with an anti-myc (for VLDLR) (second panel). Western blot analysis showing comparable expression levels of the two FE65 constructs and VLDLR (third and fourth panel). [file 1750-1326-7-9-S1.PDF]

# APP processing

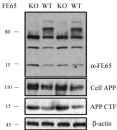

Supplementary Fig.2

Supplement: Additional file 2 — Figure S2. FE65 Knockout mice have increased APP processing. 13 month old FE65 knockout mice or wild-type littermates were immunoblotted with FE65, C1/6.1 (for APP), and β-actin. FE65 knockout mice had increased levels of full length APP and APP CTF compared to wild type littermates. [file 1750-1326-7-9-S2.PDF]
